# Supplementary material for: Neural Effects of One’s Own Voice on Self-Talk for Emotion Regulation
Source: Brain Sci. 2024 Jun 26;14(7):637. doi: 10.3390/brainsci14070637 (PMC11274574; doi:10.3390/brainsci14070637)
Supplement: Supplementary file 1 [file brainsci-14-00637-s001.zip › brainsci-3055087-supplementary.pdf]

[Supplementary material]

Sentences for each of the three conditions used in the fMRI experiment

In the experiment, these were given in Korean with similar sentence lengths, but are listed in the English translation for ease of understanding.

Self-affirmation

1. I sincerely support my friends to do well.
2. I am a man who deserves a happy life.
3. I am a man who can truly appreciate the present.
4. I believe I will succeed one day.
5. I can solve any difficulties wisely.
6. I can properly control my feeling and thoughts.
7. I can wait carefully for an important chance.
8. I do what I have to do today with pleasure.
9. I believe that I can always achieve my dream.
10. I treat people around me with a warm heart.
11. When I think about my future, I feel hopeful.
12. When I think about my life, I'm satisfied overall.
13. Even if I fail, I can overcome it.
14. There is a willingness within me to develop my abilities satisfactorily.
15. It's always fun to try new things.
16. There is potential and promise in me.
17. There is always gratitude in my heart.
18. The more difficult the situation is, the calmer I become.
19. There are many kind and grateful people around me.
20. There are many people who can help me when I'm in trouble.

Cognitive defusion

1. I put thoughts on the leaves and send them down to the river.
2. I see the train with experience moving away.
3. My negative emotions collide like waves and disappear.
4. I write down the struggle on a paper ship and float it away.
5. I scatter the tears of hurt in the calm waves.
6. I send off the crushing burden of life on a truck.
7. I write down thoughts on a paper airplane and send it away.
8. I put haunting thoughts into the bubbles and pop them.
9. I put all my anger, fury and stress on a cloud and let them go.
10. I blow away the tension like a dandelion seed.
11. The heart of regret is a talkative person who likes to meddle.
12. A bad idea is just a bait thrown by an angler.
13. You don't have to believe everything your stray thoughts say.
14. The fight against negative thoughts is an unnecessary fight.
15. Thoughts in the head are neither good nor bad.
16. Obsession is not a friend, but a strange passerby.
17. Worry is a constantly noisy storyteller.
18. Hard work is like a briefly pouring shower.
19. Disturbance of mind is like the waves of the ebb and flow.
20. The mind of tension is like an ice that will melt away.

Neutral

1. I seal opened tea and store it in the refrigerator.
2. I should close the Internet window for security.
3. I expect to be sunny from today to tomorrow.
4. I should use a hand sanitizer before eating.
5. I should tap the transport card when getting on and off the bus.
6. I must bring ID when picking up the ticket on-sit.
7. I can order this product as a set or as a single product.
8. I should keep valuable items at risk of loss in private.

9. I must prepare the minutes by the day after the meeting.
10. I can pay by credit card or cash.
11. Products that have been cleaned are safe to eat.
12. Documentation should be completed as soon as possible.
13. Here are the ways to enjoy the drink deliciously.
14. The marketing team is in charge of promoting various media.
15. An email notification will be sent again when the schedule is confirmed.
16. The departure time of the plane is continuously delayed.
17. The presenter must be punctual for the presentation.
18. A new member signs up by accessing the website.
19. Customers' understanding and cooperation might be required.
20. Everyone has the right to privacy.
